# Serial Multiple Mediation of Professional Identity, and Psychological Capital in the Relationship Between Work-Related Stress and Work-Related Well-Being of ICU Nurses in China: A Cross-Sectional Questionnaire Survey

**DOI:** 10.3389/fpsyg.2020.535634

**Published:** 2020-12-22

**Authors:** Cuiping Hao, Lina Zhu, Suzhen Zhang, Shan Rong, Yaqing Zhang, Jiuhang Ye, Fuguo Yang

**Affiliations:** ^1^Department of Critical Medicine, Affiliated Hospital of Jining Medical University, Jining, China; ^2^School of Nursing, Qingdao University, Qingdao, China

**Keywords:** professional identity, psychological capital, serial-multiple mediation model, work-related stress, work-related well-being

## Abstract

This study aimed to investigate the serial-multiple mediation effect of professional identity, psychological capital (PsyCap), work-related stress, and work-related wellbeing among intensive care unit (ICU) nurses in China. The cross-sectional survey was conducted from January 2017 to May 2017 in two Grade III A general hospitals (with more than 2000 beds) in Jining, Shandong Province, China. Cluster sampling was used to recruit participants from the two hospitals. A total of 330 ICU nurses participated in the study. The nurses’ work stress scale, Chinese nurse’s professional identity scale, the PsyCap questionnaire, and Chinese work-related wellbeing scale were used to collect the data. Descriptive analysis, independent-samples *t-*test, one-way analysis of variance, Pearson correlation analysis, linear regression analysis, and structural equation modeling were used to analyze the data (*P* < 0.05 was considered statistically significant). The average score for the work-related wellbeing of ICU nurses was 85.91 ± 13.94. Work-related stress, professional identity, and PsyCap correlated significantly with work-related wellbeing. The major predictors of work-related wellbeing were PsyCap, work-related stress, professional identity, and monthly salary. The serial-multiple mediation effects of professional identity and PsyCap in the relationship between work-related stress and work-related wellbeing were statistically significant. Positive professional identity and PsyCap were sequentially associated with decreased work-related stress, which in turn was related to increased work-related wellbeing among ICU nurses. Therefore, this study aims to explore the impact of ICU nurses’ work-related stress on work-related wellbeing, as well as the mediating effect of professional identity and PsyCap. It is hoped that hospital care managers will pay attention to the mental health of ICU nurses, increase their professional identity, and reduce work-related stress to improve the quality of the ICU nursing service and stabilize nursing work.

## Introduction

Nurses in the intensive care unit (ICU) are under more continuous stress than nurses in other general wards because of their high acuity, their heavy workload, the accidental death of patients, and perceptual conflicts with patients or other staff members (Liu, 2012; Liu et al., 2018). Intense workload leads to heavy stress among ICU nurses, causing them to lose interest in their work and become more open to leaving the workplace (Kim and Yeom, 2018). A previous study suggested that ICU nurses experienced a high level of burnout, and in the same study, a correlation between the degree of burnout and spiritual wellbeing was established (Yazdannik et al., 2012). This finding indicated that work-related stress affected the work-related wellbeing of ICU nurses.

Professional identity is a form of social identity and includes deep insight into professional performance, which also includes the establishment of professional values and goals that are widely accepted by staff (Chai, 2012). The professional identity of a nurse primarily entails the recognition of his/her goals and social values; factors in the nursing profession, including personal experience and dignity; and other factors in his/her self-image as a nurse (Hao et al., 2014; Pardanjani et al., 2014; Heldal et al., 2019). Positive professional experience in the workplace is very important for accomplishing multiple tasks, becoming more productive, experiencing job satisfaction, and improving retention in the profession (Lakanmaa et al., 2012; Mousazadeh et al., 2018). ICU nurses with a better professional identity respond to patient care more quickly and with greater interest and show respect for their patients (Culbertson et al., 2010; Worthington et al., 2013). Additionally, previous studies showed that professional identity reduced job-related burnout, accelerated interest at work, improved work enthusiasm and quality of care, and kept the nursing team together (Deng et al., 2013; Duan et al., 2017).

The rising awareness of positive psychology has recently made researchers realize the unmet need for this approach. Positive psychological capital (PsyCap) is an important positive psychological quality that can effectively predict individual job performance, job satisfaction, and burnout rate by influencing individual subjective wellbeing and social benefits (Hmieleski and Carr, 2007; Ali and Ali, 2014; Kappagoda and Alwis, 2014; Kwok et al., 2015; Youssef-Morgan and Luthans, 2015). One study found that work-related wellbeing was significantly influenced by daily positive emotions and personal life satisfaction (Zhou et al., 2018). Changes in work-related wellbeing are predicted by measuring PsyCap (Liu, 2017; Zhou et al., 2017). Recently, the PsyCap of nurses has caught the attention of many research investigators, and many existing studies are underway. PsyCap can improve the enthusiasm and work input of nurses, increase the sense of organizational mission, reduce work pressure and burnout, and improve the quality of care (Luthans and Jensen, 2005; Santos et al., 2017; Jarden et al., 2018; Kim and Yoo, 2018).

Many previous studies have explained theoretical perspectives on job wellbeing (Deci and Ryan, 2008; Page and Vella-Brodrick, 2009; Fisher, 2014; Laine and Rinne, 2015). The degree of perceived wellbeing varies with profession and associated stress level, and unique characteristics are associated with perception (Cai et al., 2018). Jarden Rebecca et al., in their study on ICU nurses in New Zealand, identified a unique concept of work-related wellbeing. Although workload and work-life balance contributed significantly to quality of life, feelings of being valued, respected, and supported were considered more important. In another study, professional happiness was defined as “the psychological and pleasant experience of the personalized work goal” based on previous theories (Zou et al., 2015). Work wellbeing is a dynamic process that requires sustained efforts and investment by organizations and individuals. It includes a wide range of structures, such as work input, flow experience, job satisfaction, and positive work emotions. The study also proposed a multilevel dynamic formation mechanism model of work-related wellbeing and established that organizational practice and organizational characteristics had a positive impact on work-related wellbeing. The influence also affected work-related wellbeing indirectly through the match of the human environment (Zou et al., 2015). A nurse’s professional wellbeing is influenced by many factors, such as his/her personal life, work environment, and work ethics (Estiri et al., 2016).

The framework of this study is mainly influenced by the existing theoretical models in psychology and nursing research, which indicate that working environment and stress characteristics affect nurses’ work wellbeing (Rochefort and Clarke, 2010; Van Bogaert et al., 2014; Pahlevan et al., 2018). PsyCap can improve employees’ work enthusiasm and work wellbeing, and the characteristics of working environment and work stress indirectly affect employees’ work enthusiasm. The more an employee’s sense of professional identity and belonging perceive PsyCap, it will indirectly affect work wellbeing. Therefore, we propose the following hypotheses: (1) work-related stress, professional identity, and PsyCap have different effects on work-related wellbeing, (2) professional identity serves as a moderator between work-related stress and work-related wellbeing, (3) PsyCap serves as a moderator between work-related stress and work-related wellbeing, and (4) professional identity and PsyCap serves as moderators between work-related stress and work-related wellbeing.

The present study aimed to test the aforementioned hypotheses and evaluate the relationship between work-related stress, professional identity, PsyCap, and work-related wellbeing. It also aimed to explore the factors associated with work-related wellbeing among ICU nurses in Chinese tertiary hospitals. The findings suggest that the ICU nursing team should be stabilized, and the quality of nursing services should be further improved to provide a reference basis for hospital managers and improve the work-related wellbeing of ICU nurses.

## Materials and Methods

### Measurement

A quantitative questionnaire survey was developed to meet the objective of this study, and written informed consent was obtained from all participants before they completed the questionnaire. The questionnaire was divided into five sections. The first section collected basic sociodemographic information. The four remaining sections were designed to assess the relationship among the independent variables of this study: work-related stress, the mediating variable “professional identity and PsyCap,” the dependent variable “work-related wellbeing,” and the mediating effect of “professional identity and PsyCap.” Then, full ethical clearance was obtained from Affiliated Hospital of Jining Medical University Research Ethics Committee (2016C005). All participants provided their written consent before completing the questionnaires. The data were collected and analyzed anonymously.

### Data Collection

The cross-sectional survey was conducted from January 2017 to May 2017 in two Grade III A general hospitals (with more than 2000 beds) in Jining, Shandong Province, China. Cluster sampling was used to recruit participants from the two hospitals. A total of 330 ICU nurses participated in the study. Everyone received a questionnaire and 314 valid responses (the effective response rate was 95.0%). All participants had been nursing in an ICU for more than 1 year.

## Instruments

### Measurement of PsyCap

The PsyCap questionnaire was adopted from a previous study by Luthans and Jensen (2005). A total of 20 items are categorized into four different dimensions as follows: items 1 through 6 are categorized as the “self-efficacy” dimension, items 7 through 12 as the “hope” dimension, items 13 through 17 as the “resilience” dimension, and items 18 through 20 as the “optimism” dimension. Each item is rated on a scale of 1–6, where 1 corresponds to strongly disagree and 6 to strongly agree. The Chinese version of the nurses’ psychological capital questionnaire was revised following Luo Hong’s translation method and presented acceptable test-retest reliability (range: 0.72–0.89) (Luo and Hao, 2010). Luthans and Jensen (2005), the original author of the PsyCap questionnaire, found that Cronbach’s α coefficient ranged from 0.77 to 0.93. Hu et al. (2015), in a study including 507 ICU nurses, indicated that the Chinese version of the PsyCap was convenient to administer, easily comprehensible, and took approximately 5 min to complete.

### Chinese Nurses’ Work-Related Stress Scale

A work-related stress scale for Chinese nurses based on national conditions was compiled by Li and Liu (2000) based on the nurses’ work-related stress scale created by Gray-Toft and Anderson (1981). The scale includes 35 items and five dimensions: nursing work and professional aspects (7 items), workload and time distribution (5 items), working environment and equipment (3 items), patient care (11 items), and management and interpersonal relationships (9 items). Each dimension is scored on a scale of 1–4, where 1 corresponds to “never” and 4 to “every day.” The Chinese version of the Chinese nurses’ work stress scale introduced by Yu (2007) showed a good internal consistency coefficient (α = 0.94), content validity (0.95), and construct validity (KMO = 0.928) and was validated in a sample of Chinese nurses. The Chinese nurses’ work-related stress scale was previously shown to be suitable for use with Chinese nurses, as it had excellent internal reference validity (Cronbach’s α range: 0.83–0.98) (Liu et al., 2016). The Chinese nurses’ work stress scale requires approximately 5–10 min for completion.

### Nurses’ Professional Identity Scale

The professional status scale for Chinese nurses was designed and developed by Liu et al. (2009a) based on interviews, expert consultations, and literature reviews. The nurses’ professional identity scale comprises five dimensions: professional cognition assessment (9 items), professional social support (6 items), professional social skills (6 items), professional coping with frustration (6 items), and professional self-reflection (3 entries). Each item is scored on a scale of 1–5, where 1 corresponds to “very inconsistent” and 5 to “very consistent.” The scale score is the sum of all items, and the total score ranges from 30 to 150. A high scale score indicates a high level of professional identity (Liu et al., 2009b). The scale showed a good internal consistency coefficient (α = 0.938) and validity (KMO = 0.930). Lin et al. (2016), in a study of 166 ICU nurses, indicated that the Chinese version of the Chinese nurses’ professional identity scale was convenient to administer, easily comprehensible, and took approximately 6 min to complete.

### Nurses’ Work-Related Wellbeing Scale

The Chinese work-related wellbeing scale was designed and developed by Chen and Liu (2016) based on interviews, expert consultations, and literature reviews. It contains 19 entry items and five dimensions: benefits (4 items), interpersonal relationships (4 items), work values (5 items), managers (3 items), and job characteristics (3 items). Each item is scored using a 6-point Likert-type scale ranging from 1 to 6, where 1 corresponds to “totally disagree” and 6 to “totally agree,” and it has shown a good internal consistency coefficient (α = 0.914) and validity (KMO = 0.883) (Wang et al., 2015; Chen and Liu, 2016). Zhu (2014), in a study including 280 ICU nurses, indicated that the Chinese version of the Chinese work-related wellbeing scale was convenient to administer, easily comprehensible, and took approximately 5 min to complete.

### Demographic and Clinical Characteristics

Based on the literature review, a set of demographic variables was included. Surrogate characteristics included age, gender, years of working the ICU, position, title, and monthly income.

In addition, work-related stress was the independent variable, while work-related wellbeing was the dependent variable. The mediating variables were professional identity and PsyCap level.

### Data Analysis

Descriptive analysis, independent-samples *t-*test, and one-way analysis of variance (ANOVA) were used to describe and compare the demographic data and the distribution of work-related wellbeing. The Pearson correlation analyses of the four variables (work-related stress, PsyCap, professional identity, and work-related wellbeing) were performed using SPSS version 22.0. The method provided by Wang and Ye (2014) was adopted, and Mplus 8.3 was used to estimate the mediation effect to test the importance of the multimedia model in this study. The deviation-corrected percentile bootstrap method was used to estimate the parameters, and the 95% confidence interval (95% CI) of the parameters was estimated by drawing 5000 bootstrap samples. The PROCESS macro was performed using one independent variable (work-related stress), two mediators (professional identity and PsyCap), and one dependent variable (work-related wellbeing). The core hypothesis model tested was how work-related stress influenced work-related wellbeing via professional identity and PsyCap.

## Results

### Preliminary Analyses

Descriptive analysis, independent-samples *t-*test, and one-way ANOVA were used to describe and compare the demographic data (age, sex, marital status, years of working in the ICU, position, and monthly salary). The distribution of work-related wellbeing is shown in Table 1. Based on the basic information provided in the questionnaire survey, of the 314 ICU nurses who completed the survey, 84.7% were women and 15.3% were men. The survey results also showed that 64.8% of the respondents were between 20 and 30 years of age. In addition, 92.4% of the ICU nurses had worked for 1–10 years, and 81.2% were general clinical nurses. In terms of the educational background of the ICU nurses, 83.1% had a bachelor’s degree or higher, and 16.9% had a college degree or lower. The raw score for work-related wellbeing was 85.91 ± 13.94.

**TABLE 1:** Demographic characteristics of subjects and intention to work well-being (*n* = 314).

Category	Subcategory	N (%)	Work well-being
Gender	Male	48 (15.3%)	84.90 ± 16.77
	Female	266 (84.7%)	86.12 ± 13.39
T		0.560	
P		0.025	
Age (year)	20∼25	45 (14.3%)	86.27 ± 13.54
	26∼30	170 (54.1%)	84.61 ± 14.25
	31∼35	78 (24.8%)	86.74 ± 13.66
	>35	21 (6.7%)	92.95 ± 11.57
F		2.419	
P		0.066	
ICU Working Time (year)	1∼5	198 (63.1%)	85.13 ± 14.17
	6∼10	92 (29.3%)	86.43 ± 13.74
	11∼15	15 (4.8%)	88.60 ± 13.74
	>15	9 (2.9%)	94.11 ± 8.40
F		1.484	
P		0.219	
Position	No	255 (81.2%)	84.65 ± 14.21
	Nurse group leader	38 (12.1%)	91.24 ± 12.43
	Nursing trainer	9 (2.9%)	88.22 ± 8.57
	The head nurse	12 (3.8%)	94.67 ± 8.35
F		4.340	
P		0.005	
Department	Comprehensive ICU	122 (38.9%)	83.75 ± 12.47
	Emergency ICU	53 (16.9%)	89.00 ± 15.40
	Heart ICU	32 (10.2%)	90.50 ± 13.67
	Pediatric ICU	107 (34.1%)	85.53 ± 14.44
F		3.085	
P		0.028	
Monthly income (yuan)	3000∼4000	53 (16.9%)	84.38 ± 14.20
	4001∼5000	131 (41.7%)	83.34 ± 12.74
	5001∼6000	66 (21.0%)	84.17 ± 13.40
	6001∼7000	13 (4.1%)	99.62 ± 13.97
	7001∼8000	21 (6.7%)	90.00 ± 14.72
	>8000	30 (9.6%)	95.10 ± 12.23
F		7.393	
P		<0.001	

One-way ANOVA showed significant differences in anxiety symptoms related to sex (*T* = 0.560, *P* = 0.025), professional title (*F* = 4.340, *P* = 0.005), department (*F* = 3.085, *P* = 0.028), and monthly salary (*F* = 7.393, *P* < 0.001) but not age (*F* = 2.419, *P* = 0.066) or years of working in the ICU (*F* = 1.484, *P* = 0.219) among ICU nurses (Table 2).

**TABLE 2:** Correlations between work stress, professional identity, psychological capital and work well-being (*n* = 314).

Variables	1	2	3	4
1. Work stress	1.000			
2. Professional identity	−0.422**	1.000		
3. Psychological capital	−0.321**	0.700**	1.000	
4. Work well-being	−0.510**	0.722**	0.668**	1.000
Mean	74.30	112.46	91.85	85.91
Standard deviation	13.67	17.79	12.71	13.94

*p < 0.05, **p < 0.01, ***p < 0.001.

### Preliminary Correlation Analyses

The correlations among the respondents’ work-related wellbeing and scores for work-related stress, professional identity, and PsyCap are outlined in Table 3. As expected, the level of work-related wellbeing was negatively correlated with the participants’ work-related stress scores (*r* = -0.510, *P* < 0.001). Professional identity was positively associated with work-related wellbeing (*r* = 0.772, *P* < 0.001). The level of work-related wellbeing was positively correlated with participants’ PsyCap scores (*r* = 0.668, *P* < 0.001).

**TABLE 3:** Hierarchical regression analysis for variables work well-being to remain (*N* = 314).

Variables	B	SE	Beta	*t*	*p*
Constant term	35.699	5.589		6.388	< 0.001
Professional identity	0.386	0.038	0.493	10.117	< 0.001
Work stress	–0.237	0.038	–0.232	–6.171	< 0.001
Psychological capital	0.239	0.051	0.218	4.643	< 0.001
Monthly income	0.925	0.335	0.098	2.757	0.006

**F* = 156.613, *R* = 0.818, *R^2^* = *0.670*,Δ*R^2^* = *0.665, P* < 0.001.*

### Linear Regression Analysis and Path Analysis of Factors Related to Work-Related Wellbeing

Considering work-related well-being as a dependent variable, a univariate analysis of sex, position, monthly income, specialist nurse training certification, and other statistically significant variables was conducted. Additionally, considering PsyCap, work-related stress, and professional identity as independent variables, multiple linear stepwise regression analysis was performed. The results suggested that the professional identity, work-related stress, PsyCap, and monthly income of ICU nurses should be entered into the regression equation, which explained 66.5% of the variation, and the regression equation was statistically significant, as shown in Table 4 (*F* = 156.613, *P* < 0.001).

**TABLE 4:** Comparison of indirect effects of work stress on work well-being mediated by professional identity and PsyCap.

	Product of Coefficients		Bootstrapping 95% BC Confidence Interval (Cl)	
Effect	Point Estimate	Boot SE	BootLL Cl	BootUL Cl
Total indirect effect:X→Y	−0.300	0.038	−0.377	–0.226
Indirect effect 1:X→M1→Y	−0.225	0.039	−0.307	–0.154
Indirect effect 2:X→M1→M2→Y	−0.075	0.018	−0.116	–0.043
Indirect effect 3:X→M2→Y	−0.004	0.013	−0.033	0.018

**N* = 314. Number of bootstrap samples for bias corrected bootstrap confidence intervals: 5000. Level of confidence for all confidence intervals: 95%. X, work stress; M1, professional identity; M2, PsyCap; Y, work well-being. According to the path analysis effect decomposition principle, the model fits well. The direct effect is the path coefficient from X to Y −0.206, and the path X→M2→Y confidence interval contains 0, which is not significant. The total indirect effect is equal to the sum of the two special paths is −0.30, and the indirect effect is –0.30/(–0.206–0.30) = 0.593. That is, 59.3% of the effect is achieved through Professional Identity and PsyCap.*

### Mediation Analyses

**FIGURE 1:**
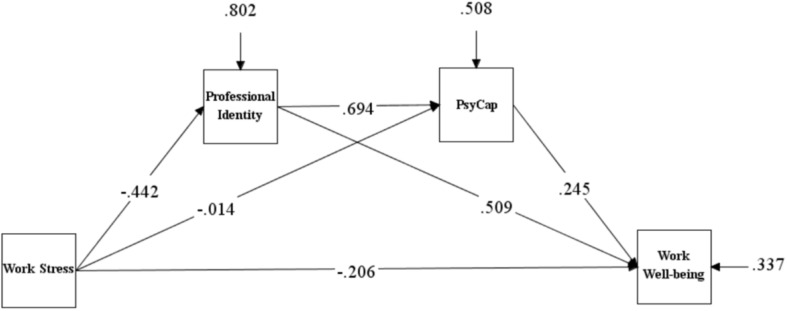
Serial-multiple mediation of professional identity and PsyCap level in the relationship between work stress and work well-being.

MPLUS 8.3 was used to test the multiple mediation model. The mediation model in which work-related stress affected work-related wellbeing through occupational identity and PsyCap fit well according to all indicators (χ^2^/*df* = 0.000, CFI = 1.00, TLI = 1.00, RMSEA = 0.000, and SRMR = 0.000) (Figure 1).

As shown in Table 3, when taking into account all variables in the tested model, the path through the single mediation factor of professional identity [point estimate = − 0.225; 95% BC CI (-0.307, -0.154)] and the path through both mediators [point estimate = − 0.075; 95% BC CI (-0.116, -0.043)] were statistically significant. However, the path through the single mediation factor of PsyCap [point estimate = − 0.004; 95% BC CI (-0.033, 0.018)] was not statistically significant. The total indirect effect was also statistically significant [point estimate = − 0.300; 95% BC CI (-0.377, -0.226)]. Therefore, the path through these two intermediaries was significant, and the indirect influence of professional identity alone was also significant, but the indirect influence of PsyCap alone was not significant.

## Discussion

In this study, the prevalence of work-related wellbeing among ICU nurses in a region of China was revealed. Furthermore, a model of the association among work-related stress, professional identity, PsyCap, and work-related wellbeing was explored by testing a serial-multiple mediation model using bootstrapping methods.

The results of this study showed that the current job happiness of ICU nurses was 4.52 out of 6, which was lower than that of new oncology nurses in China (4.65) (Zhao and Hao, 2020) but higher than that of ICU nurses in South Korea (3.16) (Kim and Yeom, 2018). The results showed that the Chinese ICU nurses had a moderately high level of work-related wellbeing, so they needed proper psychological care. This study explored the mechanism that might explain how work-related stress affects ICU nurses’ work-related wellbeing.

First, the findings suggest a strong negative correlation between work-related stress and work-related wellbeing in Chinese ICU nurses, which is in agreement with a previous study (Ke, 2017). A number of studies have confirmed that work-related stress is a negative predictor of nurses’ job satisfaction and happiness (Sun et al., 2013; Jing et al., 2016). Faced with critically ill patients, ICU nurses need to work long hours and often work overtime. Additionally, they experience considerable disorder at night, coupled with cumbersome nursing paperwork, and physical exertion when changing shifts, which puts them under tremendous pressure. Moreover, coupled with poor social support and an increased risk of injury and exhaustion, increased stress, fatigue, anxiety and emotional exhaustion, and poor health scores are more likely to affect the physical and mental health of ICU nurses, thereby affecting their wellbeing (Jones et al., 2015).

From the perspective of positive psychology, the present study tested a serial-multiple mediation model using bootstrapping methods and revealed the potential mechanism of the relationship between work-related stress and work-related wellbeing among ICU nurses. Based on the results from the serial-multiple mediation model and the contrasting pairs of specific indirect effects examined, work-related stress was found to have both direct and indirect effects on work-related wellbeing in ICU nurses via the mediation of professional identity and PsyCap. The mediation model showed that work-related stress impacts work-related wellbeing through two crucial pathways: (1) professional identity partly mediated the relationship between work-related stress and work-related wellbeing; and (2) the serial-multiple mediation of professional identity and PsyCap in the relationship between work-related stress and work-related wellbeing was statistically significant. The separate mediation effect of individual mediating variables on anxiety symptoms was statistically significant among Chinese female nurses. One of the mediating variables examined was professional identity. The results indicated that nurses who had a low level of work-related stress were more likely to have a high level of professional identity, which, in turn, led to more signs of work-related wellbeing. Nurses with a high sense of identity with ICU work are better able to alleviate work pressure, face difficulties and setbacks, and solve professional problems in their own work (Ferrell et al., 2017). However, the results for PsyCap as a separate intermediary variable were not significant. The reason for this finding might be that among the ICU nurses, the PsyCap of those with low work-related stress was not high, leading to greater work-related wellbeing.

In particular, we aimed to determine the potential mechanism underlying the relationship between work-related stress and work-related wellbeing. The results of the present study demonstrated that low work-related stress was sequentially associated with increased professional identity first and then with increased PsyCap, which in turn was related to the increase in work-related wellbeing. A large number of studies have shown that work-related stress has a negative impact on work-related wellbeing (Sun et al., 2013; Jang et al., 2019). In addition, according to a study of nurses by Wan, occupational identity was positively correlated with PsyCap (Sun et al., 2012; Wan et al., 2013). ICU nurses with higher professional identity might be more productive and therefore more satisfied and happier. In addition, compared with ICU nurses with lower professional identity, nurses with higher professional identity might have greater willingness to continue to work in the ICU and have higher PsyCap. Ultimately, this also leads to an increase in happiness at the workplace.

### Limitations

This study had several limitations. First, this study had a cross-sectional design, so it was impossible to draw credible causal conclusions. A longitudinal study needs to be conducted in the future. Second, the data for this study were collected from ICU nurses working at two public hospitals in Jining City, Shandong Province, China, and therefore cannot represent all ICU nurses. Future studies should include samples from other regions as ICU nurses in different regions may experience different working conditions or differences in the availability of resources and support.

## Conclusion

Work-related stress had an impact on work wellbeing among Chinese ICU nurses, while professional identity and PsyCap had an intermediary role in this relationship. With positive psychology and a good professional identity, ICU nurses can explore their professional interests, cope better with work pressure, improve work-related wellbeing, and thus improve the quality of ICU nursing services. Therefore, hospital nursing managers should pay more attention to the mental health of ICU nurses, improve their professional identity and PsyCap, and reduce work-related stress to improve their work-related wellbeing, stabilize the ICU nursing team, and improve the nursing quality in the ICU.

## Data Availability Statement

All datasets generated for this study are included in the article/supplementary material.

## Ethics Statement

The studies involving human participants were reviewed and approved by the Ethics Committee of Affiliated Hospital of Jining Medical University China (2016C005). The participants provided their written informed consent to participate in this study.

## Author Contributions

All authors listed have made a substantial, direct and intellectual contribution to the work, and approved it for publication.

## Conflict of Interest

The authors declare that the research was conducted in the absence of any commercial or financial relationships that could be construed as a potential conflict of interest.
